# Balance recovery stepping responses during walking were not affected by a concurrent cognitive task among older adults

**DOI:** 10.1186/s12877-022-02969-w

**Published:** 2022-04-06

**Authors:** Inbal Paran, Hadas Nachmani, Moti Salti, Ilan Shelef, Itshak Melzer

**Affiliations:** 1 Schwartz Movement Analysis & Rehabilitation Laboratory, Department of Physical Therapy, Recanati School of Community Health Professions, Faculty of Health Sciences, Ben-Gurion University of the Negev, P.O.B. 653, 84105 Beer-Sheva, Israel; 2 Scientific Head of the Brain Imaging Research Center (BIRC), Zlotowski Center for Neuroscience, Ben-Gurion University of the Negev, 84105 Beer-Sheva, Israel; 3 Diagnostic Imaging Institute, Soroka University Medical Center, Beer-Sheva, Israel

**Keywords:** Falls, Dual-task, Balance-perturbations, Compensatory-reactions

## Abstract

**Background:**

Most of older adults’ falls are related to inefficient balance recovery after an unexpected loss of balance, i.e., postural perturbation. Effective balance recovery responses are crucial to prevent falls. Due to the considerable consequences of lateral falls and the high incidence of falls when walking, this study aimed to examine the effect of a concurrent cognitive task on older adults’ balance recovery stepping abilities from unannounced lateral perturbations while walking. We also aimed to explore whether cognitive performance accuracy is affected by perturbed walking and between task trade-offs.

**Methods:**

In a laboratory-based study, 20 older adults (> 70 years old) performed the following test conditions: (1) cognitive task while sitting; (2) perturbed walking; and (3) perturbed walking with a concurrent cognitive task. The cognitive task was serial numbers subtraction by seven. Single-step and multiple-step thresholds, highest perturbation achieved, 3D kinematic analysis of the first recovery step, and cognitive task performance accuracy were compared between single-task and dual-task conditions. Between task trade-offs were examined using dual-task cost (DTC).

**Results:**

Single-step and multiple-step thresholds, number of recovery step trials, number of foot collision, multiple-step events and kinematic recovery step parameters were all similar in single-task and dual-task conditions. Cognitive performance was not significantly affected by dual-task conditions, however, different possible trade-offs between cognitive and postural performances were identified using DTC.

**Conclusions:**

In situations where postural threat is substantial, such as unexpected balance loss during walking, balance recovery reactions were unaffected by concurrent cognitive load in older adults (i.e., posture first strategy).

The study was approved by the Helsinki Ethics Committee of Soroka University Medical Center in Beer-Sheva, Israel (ClinicalTrials.gov Registration number NCT04455607, ID Numbers: Sor 396–16 CTIL; 02/07/2020).

**Supplementary Information:**

The online version contains supplementary material available at 10.1186/s12877-022-02969-w.

## Introduction

Falls among older adults can be catastrophic, as they may lead to serious injuries and medical complications such as head injuries, hip fractures and even death [1]. The majority of falls (approximately 60%) in older adults are related to inefficient recovery after an unexpected balance perturbation (e.g. slips, trips and missteps; collisions or other interactions with the environment; surface translation for instance in public transport) [2]. When balance is lost unexpectedly compensatory recovery responses are evoked in an attempt to regain balance [3], meaning to return the center of mass to the center of base of support [4]. Therefore, effective balance recovery responses are crucial to prevent falls when balance is lost unexpectedly.

Selection and engagement of balance recovery response strategies depend on the integration of many sensorimotor processes which tend to deteriorate with age [5] and, in turn, affect recovery response efficacy. For example, older adults use more steps to recover balance (i.e., multiple steps), exhibit more foot-collisions in their recovery responses, as well as *unsuccessful* balance recovery maneuvers (i.e. fall into a harness) after stance perturbations [3, 6]. In addition, older adults tend to respond with a recovery step at lower perturbation magnitudes [6, 7] and tend to fall sideways which accounts for almost all hip fractures [1]. Compared to young adults, kinematic analyses of older adults’ recovery stepping responses during walking demonstrate slower recovery step initiation time, shorter step length, and larger safety margins of stability [8].

In addition, cognitive abilities also decline with age [9, 10], especially pre-frontal functions such as executive functions and attention [10]. Studies examining interactions between cognitive and postural functions indicate that though pre-frontal cognitive resources are deteriorating, older adults tend to increase reliance on these resources for motor control tasks compared to young adults [11]. The critical role of cognitive resources in postural functions has been demonstrated in imaging studies [12] and in kinematic research applying the dual-task (DT) methodology [13–15]. DT studies allow researchers to explore cognitive-motor interference, often referred to as DT costs or DT effects [16, 17]. DT effects elucidate trade-offs between postural and cognitive tasks as well as task prioritization [16, 17], and thus allow examination of interactions between cognitive recourses and postural functions.

In DT studies with both young and older adults, the interaction between cognitive resources and balance recovery responses to stance or walking perturbations, is demonstrated by decreased performance on postural tasks, cognitive tasks, or both [18–24]. Several studies reported situations in which DT conditions did not affect cognitive or balance recovery performance to unexpected balance loss among young adults [25, 26]. Also, several studies report improved postural stability (i.e., postural sway) of young [27, 28], and older adults [28, 29] during DT conditions while standing. These inconsistent findings are due to the fact that attentional requirements vary depending on the postural and cognitive tasks [15].

The role of cognitive functions in *balance recovery responses* to unexpected balance loss has been extensively studied under perturbed stance conditions, usually in response to forward or backward external perturbations [19, 21, 23, 24, 30, 31]. Few DT studies to date have explored cognitive involvement in recovery stepping responses to perturbed walking [22, 25, 26, 32], one incorporated an exchangeable foam surface [22], two examined perturbations from special footwear [26, 32], and one used unannounced surface translations during walking [25]. Three of these studies were with young adults [22, 25, 26], two reported no interference between cognitive and balance recovery responses [25, 26], and one reported prioritization of balance recovery over cognitive performance among young adults [22]. The studies with older adults both reported prioritization of balance recovery over cognitive performance [22, 23].

Due to the considerable consequences of lateral falls [1] and the high incidence of falls when walking [2, 33], we sought to investigate the cognitive–motor interference of older adults’ balance recovery from unannounced lateral perturbations while walking. First, we aimed to examine the effect of a concurrent cognitive task on older adults’ recovery stepping abilities (i.e., single- and multiple-step thresholds, highest perturbation achieved) as well as the kinematic parameters of these recovery stepping responses. Our second aim was to explore whether cognitive performance accuracy is affected by DT conditions. Third, we aimed to examine between task trade-offs [16, 17]. Hence, to further explore relative change between single-task (ST) and DT conditions, we also examined the DT costs (i.e., DTC). Our fourth aim was to examine whether the spatiotemporal characteristics of the first recovery step were associated with perturbation magnitudes, and whether these associations were affected by cognitive load during DT conditions.

Due to natural age-related decline in cognitive and motor reserves in healthy-older adults, we first hypothesized that recovery responses would be prioritized over cognitive performance (i.e., posture-first strategy) as the postural threat is substantial and very similar to real-life situations of balance loss. This would be demonstrated by similar step thresholds and kinematic parameters of the first recovery step between ST and DT conditions. In accordance with the task prioritization model, our second hypothesis was that due to the interference effect of an unexpected balance loss, cognitive performance will be impaired. Third, we hypothesized that DTC would manifest a trade-off in favor of the postural performance, that is, no postural DTC would be found while cognitive DTC would be negative (manifesting a relative decline in DT cognitive performance accuracy). Our fourth hypothesis was that we would find a negative association between the temporal parameters of the first recovery step and perturbation magnitudes, and positive associations between perturbation magnitudes and step length and margins of stability. We hypothesized that cognitive load would not affect these associations.

## Methods

### Study design

This study is a supplementary analysis of an ongoing prospective randomized controlled trial, approved by the Helsinki Committee of Soroka University Medical Center in Beer-Sheva, Israel (ClinicalTrials.gov Registration number NCT04455607, ID Numbers: Sor 396–16 CTIL; First Posted: 02/07/2020). Analyses in this paper are based on the baseline behavioral, kinematic, and cognitive measures of this clinical trial.

### Participants

Twenty-two older adults aged 70 to 88 years (mean age 75 ± 4 years) participated in this study. Recruitment for the study was performed by advertising in retirement homes for elderly persons, and the BGU retirees committee, as well as personal contacts and word of mouth. Screening sessions were conducted to ensure that all participants were with good general health and were able to ambulate independently, without a cane. We excluded those under 70 years old, and those who used a walker. We also excluded those who reported any vestibular impairments, recurrent dizziness, any active neurological diagnosis (e.g., stroke, Parkinson’s disease, multiple sclerosis, amyotrophic lateral sclerosis, severe peripheral neuropathies), blindness, symptomatic orthostatic hypotension, respiratory diseases, active cancer treatment, total hip or knee arthroplasty, or acute lower-limb trauma in the past year. Finally, participants with a Mini-Mental State Examination (MMSE) score of < 24 were excluded as well [34]. Out of the 22 participants that were eligible for participation, one participant was excluded due to a technical issue with voice recording; another withdrew because of dizziness during the base-line assessment trial. Thus, data from 20 participants were included in subsequent analyses (see Table 1, supplementary).

### Experimental setup

**Fig. 1:**
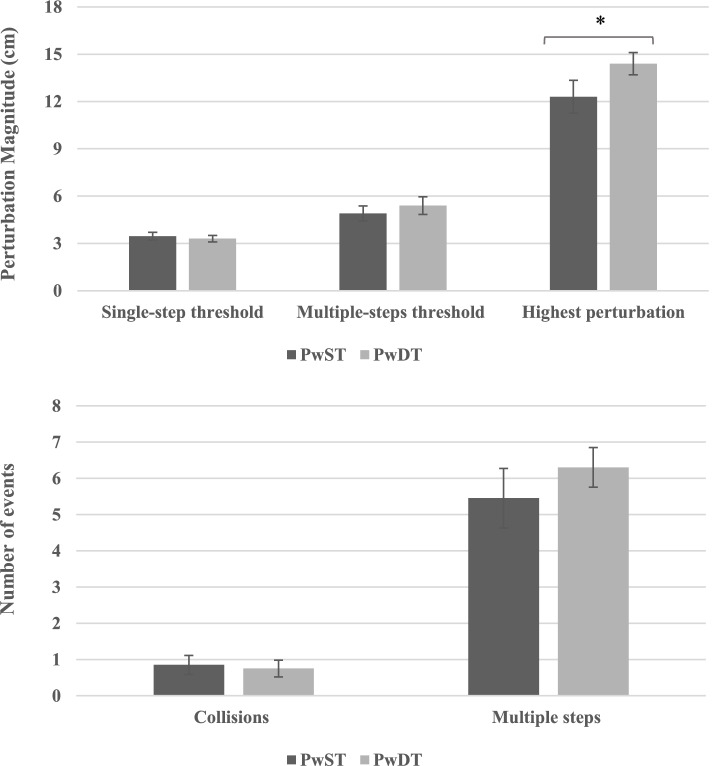
**A** Single-step and multiple-steps thresholds and the highest perturbation achieved during single-task and dual-task perturbed walking (Mean ± SEM). *p = 0.036; [**B**] The number of foot collisions and multiple steps events during single-task and dual-task perturbed walking trials. Abbreviations: *PwST* Perturbed walking condition *without* a concurrent cognitive task, *PwDT* Perturbed walking condition *with* a concurrent cognitive task

After assessing the inclusion–exclusion criteria, and signing a consent form, the participants were instructed to walk on a motor-driven treadmill (i.e., Balance Measure & Perturbation System; BaMPer System) [35] that provided right or left unannounced surface translation during walking. No handrails were mounted so that arm movements were unconstrained (Fig. 1, supplementary) no other support was provided. Instead, participants were secured in a full-trunk safety harness, designed to allow free motion but prevent ground contact (i.e., fall) in case of loss of balance.

The following conditions were studied in sequence: (1) cognitive task while sitting; (2) perturbed walking; and (3) same as (2) with performance of a concurrent cognitive task. In a prior pilot study [unpublished], we experimented with randomized testing order. We found that participants who randomly started the experiment with the most challenging condition, i.e., perturbed walking and concurrent cognitive task, tended to ask to stop the experiment. Thus, we were unable to complete the study protocol nor explore their stepping thresholds. Therefore, we gradually increased the perturbation challenges while considering the limitation of the possible learning effect. This experimental setup allowed our participants to complete the study protocol in most cases. Perturbed walking trials (with and without the concurrent cognitive task) each began with 60 s of unperturbed walking. This allowed us to examine the effect of regular walking per-se without perturbations (i.e., unperturbed walking condition) on cognitive task performance during DT walking trials; note, the effect of the cognitive task on regular walking spatiotemporal parameters is not in the scope of this study nor of the randomized controlled trial.

Participants were unexperienced with treadmill walking and thus were given 2 min to practice treadmill walking before starting the perturbed walking trials, while wearing their own comfortable shoes. The comfortable walking speed was then selected by the participants (mean velocity; 2.8 km/h). Participants were then instructed to react naturally (i.e., no instructional constraints) to a right or left unannounced surface translation while walking and try to avoid a fall. No other specific instructions were given regarding the postural performance during perturbed walking trials (i.e., with and without the concurrent cognitive task). Perturbation magnitudes systematically increased from low to high. Each perturbed walking trial included six magnitudes of unannounced surface perturbations (Table 2, supplementary) for a total of 12 perturbations (i.e., 2 directions × 6 perturbation magnitudes). This experimental set-up was based on Maki et al. [36], in which participants were exposed to two perturbation magnitudes, each magnitude included perturbations in four directions (i.e., forward, backward, lateral). We added four perturbation magnitudes, for a total of 6 magnitudes of perturbations in order to increase the sensitivity of the protocol. This non-randomized experimental set-up enabled us to explore the exact single- and multiple-step thresholds as markers of need-to-change postural strategy, from fixed-base-of-support to a change in base-of-support strategy. The order of perturbation direction was left then right, with time intervals ranging between 15 and 30 s, randomized between perturbations. Duration of the testing protocol was 5 min, but we stopped the assessment if a participant fell into the harness system or requested at any point to stop.

### Descriptive and kinematic recovery stepping responses analysis

We used the Vicon 3D motion capture system (Oxford, UK) to record and perform kinematic analyses of recovery stepping responses; this system included 16 infrared cameras operating simultaneously at 120 Hz. Camera images were mapped onto a 3D coordinate system (Vicon Nexus system software, version 2.5) using an internal direct linear transformation algorithm. Participants wore a designated whole-body suit on which 39 retro-reflective markers (radius 14 mm) were affixed at specific landmarks to track a 12-segment full body kinematic model. Anatomical landmarks were placed according to Vicon Motion System requirements [37, 38]. Two additional reflective markers were placed on the perturbation platform to track surface translation. To identify any possible postural adjustments potentially influencing the following steps, time windows extended from about 2 seconds pre-perturbation, to about 3 s post-perturbation, to measure recovery response behavior. Data exported from the Vicon system were analyzed using MATLAB code (Math Works Inc.; Cambridge, MA).

We generated 3D-motion capture stick-figure videos to identify the single- and multiple-step thresholds, as well as the highest perturbation achieved for each participant, number of feet-collisions and multiple steps events. Step thresholds were defined by the minimal perturbation magnitude after which point a single- or sequence of recovery steps consistently occurred. Highest perturbation achieved was defined as the maximal perturbation magnitude the participant reached when the trial was stopped or completed. Feet-collision events were noted if a collision between the swing and stance limb was observed in response to the unexpected perturbation. Multiple steps events were defined as perturbation trials in which more than one step was used to recover balance. The presence of the following strategies was verified offline using our MATLAB code, allowing image pauses, slow motion, and running of the image backwards and forwards. Applying similar experimental procedures Batcir et al. [6] reported excellent inter-observer reliability identifying single- and multiple-step thresholds (ICC2,1 = 0.978 and ICC2,1 = 0.971, respectively; *p* < 0.001). In addition, the following spatiotemporal recovery step parameters were measured: *Step initiation time* (ms) between the first deviation of the marker placed on the perturbation system until foot lift-off (i.e., first deviation of the marker placed on swinging leg ankle joint) more than 4 mm from the average baseline after the surface translation; *first recovery stepping duration* (ms) was calculated as the time from surface translation to foot contact on the ground completing the first step; *Step length*, calculated as the Euclidian distance (mm) for ankle markers measured from foot lift-off to foot-contact; *Step swing time* (ms) from foot-lift to foot-contact; *Margins of stability*, the distance (mm) between the extrapolated center of mass and base of support defined by the feet (i.e., ankle marker) at step initiation (i.e., foot lift-off). Larger distances represent smaller margins of stability.

### Cognitive task performance

The ‘serial sevens’ arithmetic task (e.g., 683–7 – 7 …) was chosen to induce cognitive attention-demanding load [39] and was performed while sitting and then during unperturbed and perturbed walking. MRI study conducted by Schulz et al. [39] showed that the ‘serial sevens’ arithmetic task, has the ability to efficiently distract people from another task, as it ‘required a high level of concentration. During the sitting trials, participants counted backward by seven for 5 min. In the perturbed walking trials, the cue to begin counting backward by seven was given as participants began walking and until the trial was completed, or if the participant asked to stop the experiment. The perturbed walking DT trial was about 4 min. Of note, unperturbed walking duration was about 1.5 min and was an incorporated part in the perturbed walking trials. To reduce learning effects, participants always began with a different number for the sitting and perturbed walking conditions (e.g., 683–7 – 7 … or 895–7 – 7 …. etc’). In order to compare cognitive performance across participants, all participants received the same numbers. *Participants were asked to count backward by seven as accurately as possible and try not to fall during the walking examinations, with no instructions regarding the speed of counting or motor performance*. We tracked participants performance using a Microsoft voice recorder app operated on Windows. In addition, one of the examiners tracked participants’ counting backward by seven by manually writing his/her responses. Cognitive performance accuracy was measured as the ratio between the correct answers and the total numbers counted (i.e., correct numbers + errors) under each of the three task conditions (i.e., sitting, unperturbed walking, perturbed walking).

### Dual-task cost

Dual-task costs were calculated for each task performance variable (e.g., cognitive performance accuracy) according to the traditional formula [16]:

$$\frac{\left( DT- ST\right)}{ST}\ast 100= DTC\left(\%\right)$$

In cases where a high value for a specific variable represented a reduced task performance (e.g., recovery step initiation time), a negative sign was inserted:

$$\frac{-\left( DT- ST\right)}{ST}\ast 100= DTC\left(\%\right)$$

Thus positive DTC values indicate that task performance relatively improved in DT condition, while negative DTC values indicate a worse performance in DT, relative to ST task performance [16, 17].

We calculated the DTC for the each of the first recovery step parameters as the difference between performance during perturbed-walking DT (PwDT) and perturbed-walking ST (PwST), divided by performance in PwST, for example:

$$\frac{\left( Step\ length\ in\ PwDT- Step\ length\ in\ PwST\right)}{Step\ length\ in\ PwST}$$

The DTC of cognitive performance was calculated three ways (see formulas below): (1) PwDT-UPw; the difference between cognitive performance during perturbed walking (PwDT; as the DT condition) and unperturbed walking (UPwDT; as the ST condition), divided by the performance during UPwDT; (2) PwDT-Sit; the difference between cognitive performance during PwDT (i.e., DT condition) and sitting (i.e., ST condition), divided by the cognitive performance while sitting; (3) UPwDT-Sit; the difference between the cognitive performance during UPwDT (i.e., DT condition) and sitting (i.e., ST condition), divided by the cognitive performance while sitting.

1 $$\frac{\left( Cog. performance\ in\ PwDT- Cog. performance\ in\ UPwDT\right)}{Cog. performance\ in\ UPwDT}$$

2 $$\frac{\left( Cog. performance\ in\ PwDT- Cog. performance\ in\ Sitting\right)}{Cog. perfrormance\ in\ Sitting}$$

3 $$\frac{\left( cog. performance\ in\ UPwDT- Cog. performnace\ in\ Sitting\right)}{Co\mathrm{g}. performance\ in\ Sitting}$$

### Statistical analyses

Statistical analyses were performed using Predictive Analytics Software (PASW v 26.0; Somers, NY). The Shapiro-Wilk statistic was used to test the normality of variables pooled over the sample and independently for each test condition (i.e., perturbed walking, with and without a cognitive task). We performed non-parametric statistics as most variables were not normally distributed. Statistical significance for all hypotheses was set a priori at *p* < 0.05.

In addition, we performed a complementary Bayesian null hypothesis testing analysis, using JASP software [40] to compute Bayes factors (BF; see Table 1), using default JZS priors [41, 42].

**Table 1:** Jeffreys’ scale for bayes factor interpretation

Bayes Factor	Evidence
< 1	Anecdotal: not enough evidence
1–3	Weak
3–10	Moderate
10–30	Strong
30–100	Very strong
> 1000	Decisive

To test our first hypothesis, we compared the single-step and multiple-step thresholds, highest perturbation achieved, number of foot collisions and multiple-steps events between PwST and PwDT conditions. We also examined differences in kinematic parameters between PwST and PwDT conditions at each perturbation magnitude. Scores were sign-ranked using the Wilcoxon statistic. To test our second hypothesis, the Friedman test was performed to analyze differences in cognitive performance between task conditions (i.e., sitting, UPwDT, and PwDT). To test our third hypothesis, descriptive statistics for both postural and cognitive DTCs are presented to identify trade-offs between cognitive and postural task performance (i.e., relative change in postural and cognitive performance). Finally, our fourth hypothesis was tested using curve estimation linear regression to identify associations between magnitude of perturbations and first recovery step parameters in PwST and PwDT conditions.

## Results

A total of 347 perturbation trials were performed in this study. One hundred and fifty-nine trials in the PwST condition and 188 in the PwDT condition. During PwDT trials, participants achieved significantly higher perturbation magnitudes (*p* = 0.036, Fig. 1A); in our Bayesian hypothesis testing analysis however, we found only weak evidence (BF_10_ = 2.846, see Table 1). In addition, no significant differences were found in single-step or multiple-step thresholds between PwST and PwDT conditions (Fig. 1A; *p* = 0.56, BF_10_ = 0.305; and *p* = 0.78, BF_10_ = 0.265; respectively, see Table 1 for BF description). We also compared the number of foot-collisions and multiple-step events between the PwST and PwDT conditions; here too, there were no significant differences (Fig. 1B; *p* = 0.56, BF_10_ = 0.283; and *p* = 0.26, BF_10_ = 0.441; respectively, see Table 1 for BF description). Of note, there was no difference in overall incidence of stepping responses (single and multiple steps responses) between PwST and PwDT conditions as well (χ^2^[df = 1] = 0.757, *p* = 0.384; BF_10_ = 0.114 see Table 1 for BF description).

### The spatiotemporal parameters of the first recovery step under ST and DT conditions

**Fig. 2:**
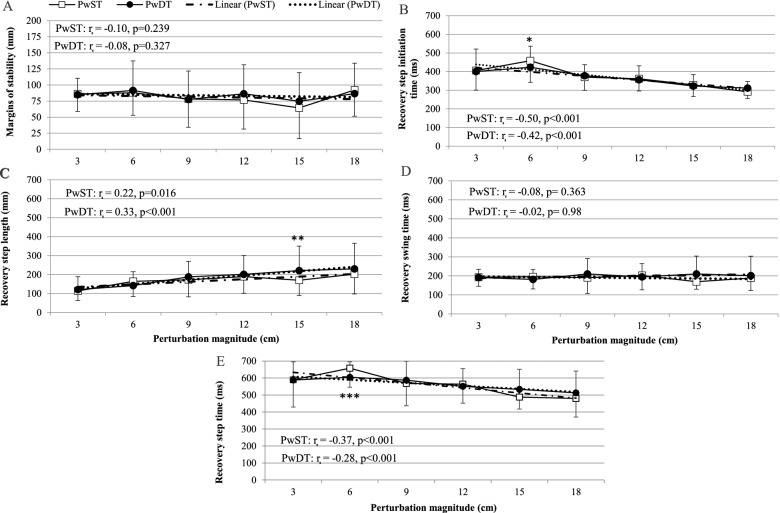
Spatiotemporal parameters (Mean ± SD) of recovery stepping responses during single- and dual-task perturbed walking. Figs. A–E: [**A**] Margins of Stability, [**B**] Reaction Time, [**C**] Step Length, [**D**] Swing Time, [**E**] Step Time. Linear regression coefficients are noted, as well as results of the Wilcoxon signed rank test; * *p* = 0.052, **p = 0.041., ***p = 0.026. Abbreviations: *PwST* Perturbed walking condition *without* a concurrent cognitive task; *PwDT* Perturbed walking condition *with* a concurrent cognitive task

We found only few significant differences in the spatiotemporal parameters of the first recovery step responses between PwST and PwDT task conditions (Fig. 2A–D, and Table 3, supplementary material). Compared to PwDT, first recovery step initiation time and the step time in PwST were found significantly slower (*p* = 0.052, and *p* = 0.026, respectively) in the small perturbation magnitude (Fig. 2B and E). Also, first recovery step length was found significantly shorter in PwST compared with PwDT (*p* = 0.041, Fig. 2C). Bayesian evidence also revealed anecdotal to weak BF_10_ values for all spatiotemporal parameters (range of BF_10_ = 0.203–2.167, see Table 1). In addition, we found that under both PwST and PwDT conditions, as perturbation magnitude increased, recovery step initiation time decreased (R^2^ = 0.230, *p* < 0.001 and R^2^ = 0.165, *p <* 0.001, respectively, Fig. 2B) and recovery step time decreased (R^2^ = 0.14, *p* < 0.001 and R^2^ = 0.06, *p <* 0.001, respectively, Fig. 2E). Also, as perturbation magnitude increased, recovery step length increased (R^2^ = 0.05, *p =* 0.016 and R^2^ = 0.124, *p <* 0.001, respectively, Fig. 2C).

### Cognitive task performance

**Fig. 3:**
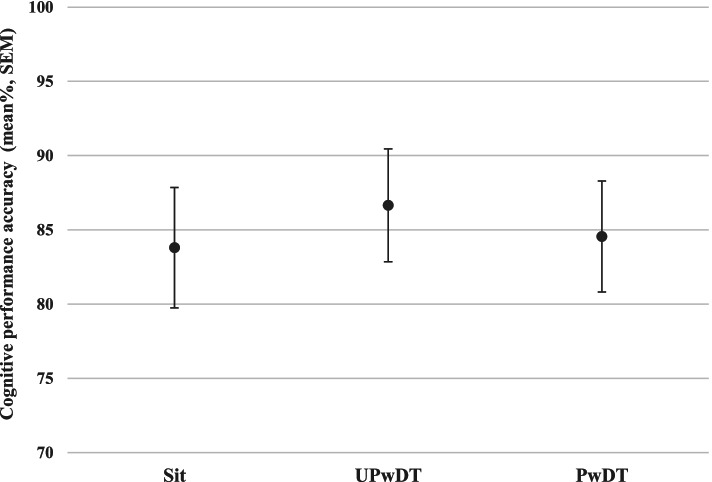
Cognitive performance accuracy (mean ± SEM) in the three task conditions (correct answers/total numbers counted, in %). Abbreviations: *Sit* Sitting task condition, *UPwDT* unperturbed walking condition with a concurrent cognitive task, *PwDT* Perturbed walking condition with a concurrent cognitive task

A trend towards significant difference was found in cognitive performance accuracy across test conditions: sitting, UPwDT and PwDT (84, 87 and 84.5%, respectively; χ^2^[df = 2] = 5.84, *p* = 0.054, Fig. 3). Post hoc analysis (Wilcoxon signed rank test) revealed no significant differences between specific task conditions (UPwDT vs. Sitting; *p* = 0.062; PwDT vs. Sitting; *p* = 0.332 and PwDT vs. UPwDT; *p* = 0.231), a tendency towards significance between UPwDT vs. sitting should be noted. In our Bayesian null hypothesis testing analysis, anecdotal Bayesian evidence (BF_10_ = 0.212, see Table 1) was found.

### Dual-task cost

Very small DTCs were found for recovery step and cognitive performance (Table 2). Step initiation time, step time and step length had small but positive mean DTC values, meaning PwDT conditions led to better performance compared to PwST (e.g., faster initiation time, faster step time and larger step length). Swing time and margins of stability, however, were negatively associated with mean DTCs, indicating relative decline (slower swing time, larger margins of stability) during PwDT compared to PwST. Mean c*ognitive* DTCs during both PwDT-Sit and UPwDT-Sit were small but positive, indicating little relative improvement in performance of the cognitive task during UPwDT and PwDT conditions compared to sitting condition. When the cognitive DTC of perturbed walking was quantified against unperturbed walking)PwDT-UPwDT(, a small negative DTC was found, indicating little reduction in cognitive performance during PwDT compared to UPwDT (Table 2).

**Table 2:** Descriptive statistics of recovery step and cognitive performance accuracy DTCs (Mean ± SEM). DTCs calculated according to Kelly et al. (2010). Note: positive DTC values indicate relative improvement in DT condition, while negative DTC values indicate worse performance in DT, relative to ST task performance. Abbreviations: *Sit* Sitting task condition, *UPwDT* Unperturbed walking condition with a concurrent cognitive task, *PwDT* Perturbed walking condition with a concurrent cognitive task

	Recovery step initiation time	Recovery step swing time	Recovery step time	Recovery step length	Margins of stability	Cognitive Accuracy PwDT-Sit	Cognitive Accuracy UPwDT-Sit	Cognitive Accuracy PwDT-UPwDT
IQR	35.22	49.89	29.22	115.09	70.85	171.07	141.46	65.93
Median	9.16	−1.54	4.37	2.59	−6.79	3.07	5.21	−2.25
Mean (%)	4.81	−2.45	4.33	9.40	−6.40	6.22	7.97	−1.49
SEM	2.50	3.00	1.96	6.72	4.47	7.13	6.14	3.33

**Fig. 4:**
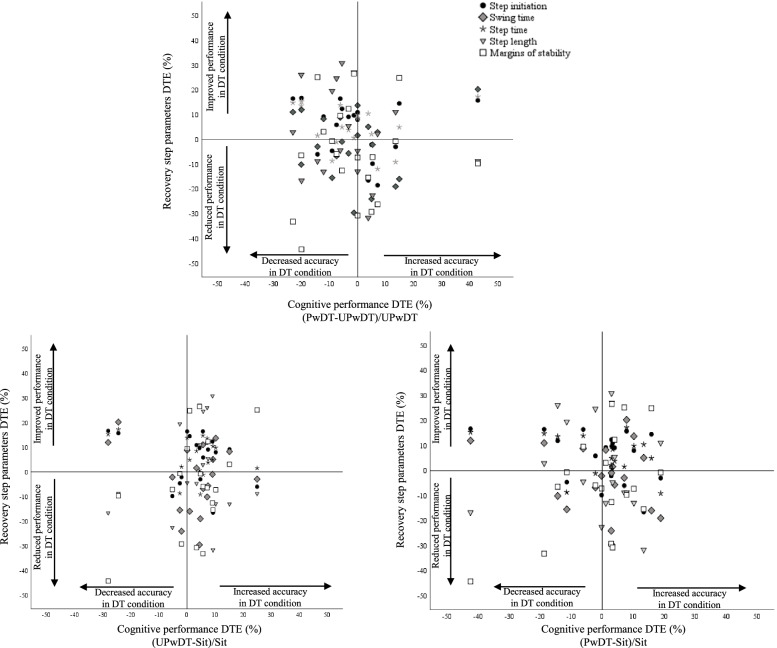
DTC (%) of the recovery stepping response parameters are plotted against cognitive performance DTC, of each participant. [**A**-**C**] Recovery stepping response DTC calculated for each parameter as the difference between the performance during PwDT and PwST divided by performance in PwST (see formula in methods). Cognitive performance DTC was calculated as follows: [A] the difference between cognitive performance during PwDT and UPwDT, divided by performance during UPwDT, [B] the difference between cognitive performance during UPwDT and sitting, divided by cognitive performance during sitting; [C] the difference between cognitive performance during PwDT and during sitting, divided by cognitive performance during sitting

A graphic approach (Fig. 4A-C) was applied to help interpret the relations between cognitive task accuracy and motor performance during PwDT conditions, presented as DTC(%). We plotted each cognitive DTC against the DTC for first recovery step parameters (recovery step initiation time, swing time, step length, margins of stability; see Fig. 4A-C). Three different tradeoffs between recovery step and cognitive task performance emerged. Fig. 4A shows a decline in cognitive performance accuracy (i.e., negative DTC), while recovery step parameters were relatively unaffected during the PwDT task condition. However, there was little relative improvement in cognitive performance during UPwDT compared to quiet sitting; motor performance was unaffected (Fig. 4B). Fig. 4C shows that cognitive performance during PwDT was similar to performance while sitting, while motor performance was unaffected.

## Discussion

This study examined the effects of concurrent cognitive load on older adults during unexpected balance perturbations while walking. In general, our hypotheses are partially supported by our findings, as they show no interference effect of concurrent cognitive task on balance recovery responses but also no significant interference effect of balance recovery on cognitive performance.

Several theoretical models that have been proposed may help explain underlying mechanisms of our findings [10, 28, 43, 44]: 1) The *capacity sharing theory* contends that since processing capacity is finite, concurrent performance of more than one task requires capacity sharing, impairing performance of at least one task [44]; 2) The *bottleneck theory* postulates, that if different tasks require similar information processing networks and cannot be processed concomitantly, a processing bottleneck occurs [44]. Consequently, concurrent task performance results in delayed performance of the secondary task and/or a slower performance of the primary task; 3) The *cross-talk theory/competition model* refers to the type of information processed rather than the operations required for task performance. Situations in which two tasks require similar inputs may create interference yet the opposite is also possible; that is, it might be easier to concurrently perform such tasks if they do not interrupt one another [44]. In the latter, no interference results and performance improvement may even occur under DT conditions; 4) the *U-shaped model* [28, 44], which postulates that when the postural and cognitive tasks are performed concurrently, postural performance can either decline or improve depending on whether the cognitive load is high or low [28, 43]; and 5) the *task prioritization model* contends that participants may prioritize the postural performance over the cognitive performance when the perceived postural threat is substantial (i.e., posture first strategy) [45].

Specifically, our findings show similar single-step and multiple-step thresholds, number of recovery step trials, number of foot collision events, multiple-step events and kinematic recovery step parameters in PwST and PwDT conditions. In addition, we found that during the PwDT trials, significantly higher perturbation magnitudes were achieved by the participants. Yet, the Bayesian hypothesis testing demonstrated only week evidence regarding this finding. This may suggests that the *task prioritization model* took place, as the participants managed to somewhat improve their postural performance by resisting more challenging perturbation magnitudes. Meaning, participants prioritized the recovery balance response over the cognitive task. We anticipated that age-related decline in balance and cognitive abilities would be reflected by poorer cognitive performance during the PwDT trials. Yet, despite the sequence of our test conditions we did not see significant change in cognitive performance under PwDT nor UPwDT conditions, compared to cognitive performance while sitting (Fig. 3). Based on the *capacity sharing theory* [44], Fig. 3 may suggest that healthy older adults in our study have sufficient attentional capacity to perform both balance recovery and continuous arithmetic tasks concurrently, i.e., no limited processing resources. A possible explanation could be that the motor-cognitive concurrent task combination in our study was too easy, generating no interference effects. This explanation, however, is unlikely. As evident by the single-step threshold, almost all participants performed a recovery step at the lowest perturbation magnitude, both in the PwST and in PwDT trials (85% of participants in PwST trials, 90% in PwDT trials). Furthermore, the multiple-step threshold indicates that most older adults needed extra recovery steps to regain their balance during their two lowest perturbation magnitudes (89.5% in PwST trials, 85% in PwDT trials). Low single-step threshold is an indicator of age-related decline in effective balance recovery [46] and multiple-steps increase risk of falling [46, 47]. This confirms that even the smallest perturbations in our protocol were sufficiently challenging for older adults. Also, the participants stated that the arithmetic task was difficult for them. Several participants in our study anecdotally reported that they were apprehensive before the start of the protocol. Several participants reported that they were embarrassed that they might not be able to perform the mathematical task without mistakes. As this was not in the scope of this study, we did not measure vigilance, anxiety, or motivation across trials, which can be considered a study limitation. However, measuring these phenomena may elucidate the mechanisms involved in recovery responses. Future research should also measure physiological arousal (e.g., Galvanic Skin Conductance; GSC), cardiac and respiratory rates. This will enable objective measurement of stress on postural recovery and vice versa.

The ‘*posture first strategy’* assumes that during DT performance, attention is shifted *towards* the postural task, leading to preservation of postural performance on the expanse of the secondary task. However, allocating attention towards the postural task can sometimes impair automatic postural processes that are occurring unconsciously [28]. For example, it has been previously reported that by shifting conscious attention away from the postural task, a more automatic postural control was possible, thus, leading to an improved postural sway under DT conditions, compared to ST performance [27, 28, 39]. It was demonstrated that when cognitive task difficulty further increased, postural performance declined, suggesting a *U-shaped interaction* of interference on balance function [28, 39]. In our study, cognitive task was similar in all conditions while postural task difficulty gradually increased, and in respect to cognitive performance accuracy, inverted U-shape interaction was apparent (*p* = 0.054, Fig. 3). Though only a trend towards a significant difference was found, cognitive performance accuracy improved during UPwDT condition compared to ST performance (i.e., in quiet sitting, *p* = 0.062) and then mildly declined during PwDT. This trend also does not support the possibility for learning effect in our cognitive results.

The graphic approach we used (Figs. 4A-C) helps to elucidate the possible trade-offs between cognitive and postural performances, which in turn can be explained by some of the theoretical models. First, Fig. 4A suggests that when PwDT is compared to UPwDT, healthy older adults used the *task prioritization model* considering that the accuracy of the cognitive task performance was somewhat reduced while recovery step kinematic parameters were relatively unaffected. Also, we noticed that when counting backwards during PwDT, most participants tended to pause their counting immediately after the perturbation occurred. These observations may support the prioritization strategy, which is commonly seen among healthy older adults who tend to shift attention away from the cognitive task towards the postural task to avoid falling [20, 45].

The small interference effect on cognitive performance shown in Fig. 4A is in partial agreement with previous studies that reported both cognitive and motor interference effects using the serial 3’s subtraction task, during perturbed stance [19, 23]. Brown et al. [19] found that during DT performance, both young and older adults counted more slowly post-perturbation compared to pre-perturbation in the cognitive task and exhibited greater center of mass distance from the margins of base-of-support when a step was executed. Using a similar protocol, Rankin et al. [23] found that the amplitude of muscle electric activity in both young and older adults was significantly reduced in DT compared to ST performance, and similarly to our results, Rankin et al. [23] found that balance recovery step initiation time was unaffected by DT conditions, however, they did not report cognitive task performance accuracy. Based on previous findings in perturbed standing protocols [19, 23], we expected that cognitive performance would be significantly impaired during the PwDT trial. The fact that we did not find such significant interference could be related to automatization of balance recovery during walking as well as the type of instructions given to our participants.

It has been previously reported that focus of attention can be affected by task instructions [16]. Instructions such as “try not to step” and “count as fast and as accurately as possible” given in previous studies, such as those by Brown et al. [19] and Rankin et al. [23], may have contributed to the interference effect they reported. In this study, we did not find a significant interference effect, this could be due to our not prioritizing one task over the other in provided instructions. For the perturbed walking task, we asked our participants to ‘walk naturally and try not to fall’, and for the cognitive task we simply instructed participants to subtract by 7 as accurate as they can until asked to stop. We did not want to impose any additional stress on the participants regarding their performance in either the postural or the cognitive task and did not want to affect natural processes of task prioritization.

Surprisingly, cognitive performance accuracy during UPwDT was even better than it was in quiet sitting (Fig. 4B). Also, when cognitive performance during PwDT was compared with quiet sitting, no cognitive motor interference was apparent (Fig. 4C). Specifically, our perturbed walking postural task was ongoing and performed after individual comfortable walking speed was selected. Since our chosen cognitive task, serial 7’s, was also continuous, it is possible that a comfortable walking speed allowed synchronization between walking speed and pace of subtraction [48]. Such synchronization might create a facilitation effect during the concurrent performance in DT trials [48], instead of interference. In an earlier study with young adults, a similar facilitating effect was reported [25]. This kind of interaction demonstrated in Fig. 4B-C can also be supported by the *cross-talk theory,* where two tasks (i.e., walking and counting backwards) do not interrupt one another and are actually easier to concurrently perform, therefore no competition or interference is created [44]. This specific combination between serial subtractions and comfortable paced treadmill walking may also be one of the limitations of this study. A less systematic cognitive task may have elicited interference effects (e.g., verbal fluency, reaction time tasks). However, we specifically selected a cognitive task which has been widely used, known to be challenging and tax cognitive resources [39] *Since the trials always occurred in the same order, this may also be a limitation of this study. It may be argued that a learning effect of the cognitive task occurred during the sitting condition, enabling the participants to perform the cognitive task better during the perturbed walking DT. However, the specificity concept of motor learning and exercise physiology argues that if learning occurred in sitting, the likelihood that the effects would immediately and directly transfer to perturbed walking task is low.*

## Conclusion

To the best of our knowledge, this is the first study to examine how older adults’ recovery stepping responses caused by unexpected surface translations are affected by attention-demanding cognitive load. Balance recovery responses to unexpected balance loss during walking were unaffected by concurrent cognitive load in older adults in this study. However, when we compared perturbed walking to unperturbed walking, cognitive performance appeared to be slightly affected by the postural challenge. It is likely that the posture first strategy was the mechanism that occurred while concurrently performing continuous cognitive and recovery from unexpected balance loss. Future studies should explore whether similar mechanisms occur in frail older adults and patient populations.

## Supplementary Information

**Additional file 1.**

**Additional file 2.**

**Additional file 3.**

**Additional file 4.**

## Data Availability

All data generated or analyzed during this study are included in this published article [and its supplementary information files] are not publicly available due to ethical considerations, but are available from the corresponding author on reasonable request.

## Abbreviations

CoM: Center of mass
DTC: Dual-task cost
ST: Single task
DT: Dual task
MMSE: Mini Mental State Examination score
Pw: Perturbed walking
UPw: Unperturbed walking
PwDT: Perturbed-walking DT
UPwDT: Unperturbed-walking DT
