# Supplementary material for: Balance recovery stepping responses during walking were not affected by a concurrent cognitive task among older adults
Source: BMC Geriatr. 2022 Apr 6;22:289. doi: 10.1186/s12877-022-02969-w (PMC8988391; doi:10.1186/s12877-022-02969-w)
Supplement: Supplementary file 1 — Additional file 1. [file 12877_2022_2969_MOESM1_ESM.docx]

**Table 1 (Supplementary).** **Demographic characteristics (mean±SD).**

| Age | 75±4 |
| --- | --- |
| Gender | M=6  F=14 |
| Weight (kg) | 72±12 |
| Hight (cm) | 163±9 |
| MMSE | 29±1 |

Abbreviations: MMSE, Mini-Mental State Examination.
