# Supplementary material for: Balance recovery stepping responses during walking were not affected by a concurrent cognitive task among older adults
Source: BMC Geriatr. 2022 Apr 6;22:289. doi: 10.1186/s12877-022-02969-w (PMC8988391; doi:10.1186/s12877-022-02969-w)
Supplement: Supplementary file 2 — Additional file 2. [file 12877_2022_2969_MOESM2_ESM.docx]

**Table 2 (supplementary)*.*** Perturbation parameters in standing and walking, by perturbation magnitude.

| Perturbation  Magnitude | Distance  (cm) | Perturbation Velocity (m/s) | Perturbation Acceleration (m/s^2^) |
| --- | --- | --- | --- |
| 1. Extra-Small | 3 | 0.11 | 0.35 |
| 2. Small | 6 | 0.22 | 0.7 |
| 3. Medium | 9 | 0.44 | 1.5 |
| 4. Medium-Large | 12 | 0.66 | 2.0 |
| 5. Large | 15 | 0.88 | 2.5 |
| 6. Extra-Large | 18 | 1.20 | 3.0 |
