# Supplementary material for: Balance recovery stepping responses during walking were not affected by a concurrent cognitive task among older adults
Source: BMC Geriatr. 2022 Apr 6;22:289. doi: 10.1186/s12877-022-02969-w (PMC8988391; doi:10.1186/s12877-022-02969-w)
Supplement: Supplementary file 3 — Additional file 3. [file 12877_2022_2969_MOESM3_ESM.docx]

**Table 3 (supplementary).** Spatiotemporal parameters (Mean ± SD, n) of recovery stepping responses during single- and dual-task perturbed walking. Abbreviations: PwST, perturbed walking condition *without* a concurrent cognitive task; PwDT, perturbed walking condition *with* a concurrent cognitive task.

| **Kinematic parameters** | **PwST** | **SD** | **n** | **PwDT** | **SD** | **n** | **p-value (Wilcoxon)** |
| --- | --- | --- | --- | --- | --- | --- | --- |
| **Step initiation time (ms) Magnitude=3 (Rt+Lt)** | 405.95 | 115.26 | 21 | 400.67 | 100.20 | 25 | NS |
| **Step initiation time (ms) Magnitude=6 (Rt+Lt)** | 458.56 | 78.69 | 36 | 423.13 | 80.89 | 40 | 0.052 |
| **Step initiation time (ms) Magnitude=9 (Rt+Lt)** | 370.00 | 67.69 | 30 | 377.71 | 77.61 | 40 | NS |
| **Step initiation time (ms) Magnitude=12 (Rt+Lt)** | 361.51 | 69.60 | 21 | 356.37 | 59.81 | 34 | NS |
| **Step initiation time (ms) Magnitude=15 (Rt+Lt)** | 328.89 | 54.63 | 15 | 323.02 | 56.70 | 21 | NS |
| **Step initiation time (ms) Magnitude=18 (Rt+Lt)** | 291.67 | 55.39 | 12 | 311.54 | 56.16 | 13 | NS |
|  |  |  |  |  |  |  |  |
| **Step time (ms) Magnitude=3 (Rt+Lt)** | 589.29 | 159.82 | 21 | 587.85 | 107.45 | 24 | NS |
| **Step time (ms) Magnitude=6 (Rt+Lt)** | 657.66 | 111.22 | 37 | 604.58 | 90.80 | 40 | 0.026 |
| **Step time (ms) Magnitude=9 (Rt+Lt)** | 568.33 | 131.30 | 30 | 587.71 | 111.72 | 40 | NS |
| **Step time (ms) Magnitude=12 (Rt+Lt)** | 563.89 | 111.38 | 21 | 551.23 | 104.27 | 34 | NS |
| **Step time (ms) Magnitude=15 (Rt+Lt)** | 488.02 | 70.84 | 16 | 533.33 | 118.17 | 21 | NS |
| **Step time (ms) Magnitude=18(Rt+Lt)** | 479.86 | 109.37 | 12 | 512.82 | 128.05 | 13 | NS |
|  |  |  |  |  |  |  |  |
| **Swing time (ms)3** | 192.86 | 47.60 | 21 | 191.32 | 43.44 | 24 | NS |
| **Swing time (ms)6** | 195.30 | 63.92 | 39 | 181.46 | 51.94 | 40 | NS |
| **Swing time (ms)9** | 190.23 | 84.14 | 29 | 210.00 | 81.63 | 40 | NS |
| **Swing time (ms)12** | 202.38 | 75.97 | 21 | 194.85 | 69.84 | 34 | NS |
| **Swing time (ms)15** | 169.27 | 39.17 | 16 | 210.32 | 94.13 | 21 | NS |
| **Swing time (ms)18** | 188.19 | 65.18 | 12 | 201.28 | 102.22 | 13 | NS |
|  |  |  |  |  |  |  |  |
| **Step length (mm)3** | 114.10 | 50.65 | 17 | 120.98 | 68.12 | 22 | NS |
| **Step length (mm)6** | 164.68 | 79.50 | 35 | 142.28 | 72.24 | 40 | NS |
| **Step length (mm)9** | 172.85 | 90.10 | 27 | 187.73 | 81.64 | 40 | NS |
| **Step length (mm)12** | 189.52 | 88.46 | 19 | 201.42 | 99.72 | 33 | NS |
| **Step length (mm)15** | 170.25 | 80.51 | 16 | 220.84 | 128.92 | 21 | 0.041 |
| **Step length (mm)18** | 202.59 | 104.88 | 10 | 230.29 | 135.16 | 13 | NS |
|  |  |  |  |  |  |  |  |
| **dBoS (mm)3** | 86.29 | 25.58 | 23 | 84.87 | 27.44 | 25 | NS |
| **dBoS (mm)6** | 87.91 | 45.79 | 39 | 91.55 | 34.97 | 40 | NS |
| **dBoS (mm)9** | 78.26 | 44.23 | 29 | 77.57 | 44.02 | 36 | NS |
| **dBoS (mm)12** | 76.79 | 44.92 | 20 | 86.38 | 45.33 | 29 | NS |
| **dBoS (mm)15** | 64.36 | 44.51 | 15 | 74.72 | 48.01 | 19 | NS |
| **dBoS (mm)18** | 92.26 | 47.24 | 9 | 86.58 | 40.56 | 12 | NS |
