# Supplementary figures and images for: Balance recovery stepping responses during walking were not affected by a concurrent cognitive task among older adults
Source: BMC Geriatr. 2022 Apr 6;22:289. doi: 10.1186/s12877-022-02969-w (PMC8988391; doi:10.1186/s12877-022-02969-w)

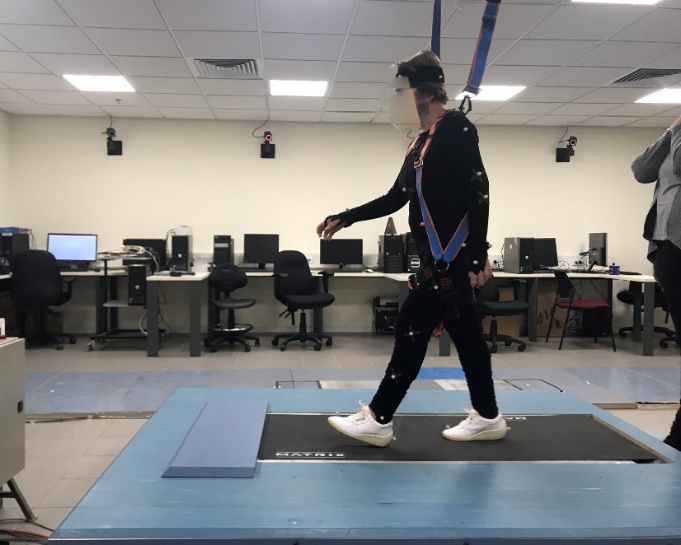
**Fig 1 (supplementary).** Experimental setup.

Supplement: Supplementary file 4 — Additional file 4. [file 12877_2022_2969_MOESM4_ESM.docx]
